# Proteomic Analysis of Plasma-Derived Extracellular Vesicles From Mice With *Echinococcus granulosus* at Different Infection Stages and Their Immunomodulatory Functions

**DOI:** 10.3389/fcimb.2022.805010

**Published:** 2022-03-10

**Authors:** Chunli Shi, Xiaojing Zhou, Wenjuan Yang, Jianwen Wu, Min Bai, Ying Zhang, Wei Zhao, Hui Yang, Atsushi Nagai, Mei Yin, Xiaoping Gao, Shuqin Ding, Jiaqing Zhao

**Affiliations:** ^1^ School of Basic Medicine, Ningxia Medical University, Yinchuan, China; ^2^ Department of Molecular Biology, Shanghai Centre for Clinical Laboratory, Shanghai, China; ^3^ College of Clinical Medicine, Ningxia Medical University, Yinchuan, China; ^4^ Department of Neurology, Shimane University Faculty of Medicine, Izumo, Japan; ^5^ Research Center for Medical Science and Technology, Ningxia Medical University, Yinchuan, China; ^6^ Ningxia Institute of Medical Science, Yinchuan, China; ^7^ Ningxia Key Laboratory of Prevention and Control of Common Infectious Diseases, Yinchuan, China; ^8^ Department of Respiratory Medicine, General Hospital of Ningxia Medical University, Yinchuan, China; ^9^ Department of Otolaryngology Head and Neck Surgery, General Hospital of Ningxia Medical University, Yinchuan, China; ^10^ Department of Medical Laboratory, School of Clinical Medicine, Ningxia Medical University, Yinchuan, China

**Keywords:** extracellular vesicles, *Echinococcus granulosus*, immunomodulatory functions, proteomics, host–parasite interactions

## Abstract

The globally distributed cystic echinococcosis (CE) is caused by the larval stage of *Echinococcus granulosus* (*E. granulosus*), a cosmopolitan and zoonotic disease with potentially life-threatening complications in humans. The emerging roles for extracellular vesicles (EVs) in parasitic infection include transferring proteins and modifying host cell gene expression to modulate host immune responses. Few studies focused on the host-derived EVs and its protein profiles. We focused on the EVs from mouse infected with *E. granulosus* at different stages. ExoQuick kit was used for isolating EVs from mouse plasma and ExoEasy Maxi kit was used for isolating protoscolex culture supernatant (PCS) and hydatid cyst fluid (HCF). Firstly, EVs were characterized by transmission electron microscopy (TEM), nanoparticle tracking analysis (NTA) and immunoblot. Secondly, the proteins of plasma EVs were identified using liquid chromatography-tandem mass spectrometry (LC–MS/MS). The resulting LC–MS/MS data were processed using Maxquant search engine (v 1.5.2.8). Tandem mass spectra were researched against the mice and *E. granulosus* proteins database in the NCBI. The differentially expressed proteins are performed by proteomic label-free quantitative analysis and bioinformatics. Thirdly, *in vitro* experiment, the results of co-culture of plasma EVs and spleen mononuclear cells showed that 7W-EVs can increase the relative abundance of regulatory T (Treg) cells and IL-10. We further verified that EVs can be internalized by CD4^+^ and CD8^+^ T cells, B cells, and myeloid-derived suppressor cells (MDSC). These results implied host-derived EVs are multidirectional immune modulators. The findings can contribute to a better understanding of the role of host-derived EVs which are the optimal vehicle to transfer important cargo into host immune system. In addition, we have found several important proteins associated with *E. granulosus* and identified in infected mouse plasma at different stages. Furthermore, our study further highlighted the proteomics and immunological function of EVs from mouse infected with *E. granulosus* protoscoleces at different infection stages. We have laid a solid foundation for the role of EVs in cystic echinococcosis in the future research and supplemented a unique dataset for this *E. granulosus.*

## Introduction

Cystic echinococcosis (CE) is a chronic zoonotic helminthic disease with a worldwide distribution. It is a highly endemic and serious disease causing a severe public health issue in western China (Wen et al., 2019). CE is an easily ignored disease because *Echinococcus granulosus* (*E. granulosus*) metacestodes have a complicated life cycle, and the transmission and development of *E. granulosus* mainly depends on the spread of the final host (dog, wolf and so on) and intermediate host. It is worth noting that humans are not usually directly involved in the transmission of CE and are not final intermediate hosts for *E. granulosus* (Woolsey and Miller, 2021). However, the main reason of infection is that humans usually get along with sheep and dogs, while dogs infected with *E. granulosus* usually carry eggs in their fur, which is easy to be eaten by humans, thus causing the disease (Tamarozzi et al., 2020).

The occurrence of hydatid disease is characterized by the development of slow-growing hydatid cysts that may not be detected for several months or even many years after the initial infection has occurred (Cucher et al., 2016). The clinical symptoms of CE are mild during the early stage of infection result from the cysts grow slowly. Thus, it is still difficult to diagnose before the cyst formation based on current imaging techniques and laboratory methods (Siles-Lucas et al., 2017a). Following when the cysts are large enough, they can cause allergic symptoms of urticaria, edema, respiratory symptoms, and anaphylactic shock (Vuitton, 2004). Moreover, it is well known that the development of hydatid cysts has a significant in parasite persistence during chronic CE. It is known from the literatures that protoscoleces develop into hydatid cysts within 20 weeks (Diaz, 2017). Based on the life cycle of *E. granulosus*, the cysts are not yet formed in 7-weeks post-infection mouse (Rogan and Craig, 1997). The hydatid cysts build a barrier that protect from the host immune response and contributes to the immune escape of the parasite (Santos et al., 2016). To date, few studies have paid attention to the changes of immune response in host before and after the formation of cysts.

In recent years, extracellular vesicles (EVs), which are able to carry proteins, lipids, and nucleic acids, have gradually emerged the field of vision of researchers (Pegtel and Gould, 2019). EVs from microorganisms (Oliveira et al., 2010; Vargas et al., 2015) and parasite have been well recognized as mediators of intercellular communication through EVs incorporated by recipient cells (Montaner et al., 2014; Torrecilhas et al., 2020). *Leishmania*-infected macrophages can release extracellular vesicles promoting lesion development (Gioseffi et al., 2020). *Silverman* and *Reiner* have proposed that *Leishmania* is capable of secreting both exosomes and plasma membrane blebs, as mammalian cells do, suggesting that *Leishmania* EVs secreted upon initial infection are capable of delivering effector cargo to naïve target cells wherein the cargo primes host cells for infection by interfering with host cell signaling pathways (Silverman and Reiner, 2011). Up to now, many researchers have focused on parasite-derived EVs (Walker et al., 2004; Nono et al., 2012; Montaner et al., 2014), while there is only a small body of literature have reported the hosted-derived EVs. (Siles-Lucas et al., 2017b; Wang W. et al., 2019; Zhou et al., 2019)

Recently, several studies have contributed to the proteomic analysis of *E. granulosus*. Siles-Lucas et al. first succeeded in isolated EVs from fertile hydatid cyst fluid (HCF) and purified and characterized the EVs during *E. granulosus* metacestode infection (Siles-Lucas et al., 2017b). In addition, other researches have highlighted larger differences in the hydatid fluid protein profiles between fertile and infertile cysts from *E. granulosus* (Santos et al., 2016). Our team previously described a proteomic analysis of EVs isolated from the serum of healthy donors and CE patients and found that some proteins are exclusively present in the EVs from the serum of CE patients, which provided new insight for the diagnosis of CE (Wang et al., 2019). Moreover, our previous proteomic analysis of EVs in protoscolex culture supernatant (PCS) and hydatid cyst fluid (HCF) of CE patients revealed an inhibitory effect of PCS-EVs on T-cell proliferation and found parasite-derived proteins and host-origin proteins in hydatid cyst fluid (HCF)-EVs (Zhou et al., 2019), which improved our understanding of EVs involving to the communication between parasite and host. We know that the development of protoscoleces grow into cysts always need take a long time, while the changes of EVs at different stages of echinococcosis development are not clear. It is well known that EVs not only act locally during CE but also circulate systemically through the vasculature and lymphoid systems (Caby et al., 2005; Del et al., 2016). Moreover, systematic and dynamic studies of the changes of the different proteins and immunomodulatory functions of EVs from host infected with *E. granulosus* at different stages are still lacking.

In present study, we use intraperitoneal infection mouse of *E. granulosus* protoscoleces experimental model. On the one hand, protein annotation and functional enrichment were performed for the identified proteins of plasma EVs from mice infected with *E. granulosus* at different stages. On the other hand, the *in vitro* co-cultural experiments were used to discover EVs effect on immune cells. Hence, the aim of this study was to observe the changes of protein expression in plasma EVs from host infected with *E. granulosus* at different stages, and to explore the immune regulated function of proteins of host-derived EVs in the development of *E. granulosus.*

## Methods

### Ethics Statement

The animal study was agreed with the commendations in the Guide for the Care and Use of Laboratory Animals of the Ministry of Science and Technology of the People’s Republic of China. All animal and human experiments in this study were approved by the Ningxia Medical University Medical Ethical Committee (permit number: 2021-846 for animal, 2019-62 for human) and were conducted strictly according to the guidelines.

### Experiment Animals

Specific-pathogen free (SPF) female mice, 6- to 8-week-old BALB/c (18–22 g), were bought from the Beijing Weitonglihua Laboratory Technology Co., Ltd. (Qualification Certificate: SCXK Beijing 2016-0006). A total of 25 mice (5 mice per group) were used to infect *E. granulosus* protoscoleces. Twenty five healthy mice (5 mice per group) were used to isolate spleen mononuclear cells in co-cultural experiment. Mice were kept under controlled SPF conditions. They were maintained with sterile drink water and food.

### Isolation of Protoscoleces and Infection of Mice

*E. granulosus* hydatid cysts were obtained from CE patients at the general hospital of Ningxia Medical University. Informed consent was obtained from the patients for publication of these case reports. We collected protoscoleces from fertile hydatid cysts from patients with CE as previously described (Zhou et al., 2020). Briefly, the intact hydatid cysts were washed three times with sterile phosphate-buffered saline (PBS) (Biological Industries, Israel) to remove impurities. We collected the liquid in hydatid cysts using a syringe and transferred the liquid into 50 ml centrifuge tubes to collect protoscoleces by natural precipitation for 30 min in ice. Next, protoscoleces were washed three times with PBS. We determined the viability of the protoscoleces by examining their motility characteristics and structural integrity, as shown in **Supplementary Figure 1A**.

A total of 2,000 live protoscoleces in 200 μl of PBS are used for intraperitoneally injected to every *E. granulosus* infected mouse (5 mice per group) and only 200 μl of PBS was used for uninfected mouse (n = 5). For each experiment, the mice were anesthetized and sacrificed by cervical dislocation at 7 W, 20 W post infection and uninfected mice (0 W).

### Isolation and Purification of EVs

Approximately 1 ml of blood samples were collected from per mouse at different infection stages with EDTA as anticoagulant. Approximately 250 μl of plasma were isolated from every mouse at 2,000×*g* for 15 min in Megafuge 8R centrifuge (Thermo Scientific, Germany) with rotor MicroClick 24 × 2. We isolated and purified the EVs from mouse plasma using the ExoQuick kit (SBI, USA), as described previously (Lee et al., 2018), according to the instructions of the manufacturer. Briefly, 2 μl of thrombin was added to 0.25 ml of plasma to a final concentration of 5 U/ml, and then the samples were mixed, incubated at room temperature for 5 min, and centrifuged at 2,000×*g* for 5 min. The supernatant was transferred to a new clean tube. The serum-like supernatant was centrifuged at 3,000×*g* for 15 min to remove cells and debris. Then 250 μl of supernatant was transferred to a sterile tube and the 63 μl of ExoQuick Exosome Precipitation Solution was added to the plasma. Samples were mixed, incubated for 30 min at 4°C, and then centrifuged at 1,500×*g* for 30 min. The supernatant was aspirated. The exosome pellet was suspended in 200 μl of sterile PBS and stored at −80°C or directly used for analysis. We used the bicinchoninic acid assay (BCA) (Thermo Fisher) to determine the protein concentration of the EVs according to the instructions of the manufacturer.

Nine mice were used to isolate plasma EVs (uninfected mice, n = 3; infected 7 weeks, n = 3; infected 20 weeks, n = 3), 3 mice per groups were treated as individual replicates, the mice were sacrificed and their plasma EVs were isolated at different infection times, and store at −80°C. Finally, 100 μg proteins of each sample were used for proteomic analysis and the rest was used for *in vitro* co-culture experiment. To increase comparability between samples, 9 samples were collected and sent together to PTM BioLab to proteomic analysis at the same time.

Hydatid cysts fluid (HCF) and protoscolex culture supernatant (PCS)-EVs stored at −80°C was obtained from our previous research (Zhou et al., 2019). The isolated EVs were isolated by exoEasy Maxi kit (Qiagen, American) according to the instructions of the manufacturer, as our previous paper described (Zhou et al., 2019).

The endotoxin activity of isolated EVs was determined using chromogenic endotoxin quant kit according to the instructions of the manufacturer (thermo scientific).

### Transmission Electron Microscopy (TEM)

We characterized EVs by transmission electron microscopy (TEM). About 25 μl of EVs was spotted on a formvar-coated 200-mesh copper grid and fixed in 2% paraformaldehyde acid for 2 min. We recorded the images on a HITACHI-H7650 (Tokyo, Japan) at 60 kV.

### Nanoparticle Tracking Analysis (NTA)

We measured the particle sizes and concentration of EVs by nanoparticle tracking analysis (NTA) with a zeta view instrument (Particle Metrix) and the corresponding software.

### Western Blot Analysis

We separated 20 μg of exosomal proteins by 10% SDS-PAGE for 90 min at 120 V. The proteins were transferred onto polyvinylidene fluoride membranes (Merck Millipore). Next, we incubated the membranes in blocking solution (5% milk in PBS containing 1% Tween-20) for 1 h at 20°C and then incubated with rabbit monoclonal antibodies against mouse CD9 (1:1,000) (SBI, USA) and CD63 (1:1,000) (SBI) to detect EVs markers. We used monoclonal rabbit anti-mouse β-actin (1:1,000) (Bioss, China) as an internal control. The membranes were washed and subsequently incubated with horseradish peroxidase-conjugated goat anti-rabbit (1:3,000) (Abcam, United Kingdom) for 1 h at room temperature. Finally, we visualized the protein bands using the Piece Fast Western Blot Kit and ECL Substrate (Thermo Scientific), and analyzed the bands using Image Lab software (Bio-Rad, USA).

### Liquid Chromatography-Tandem Mass Spectrometry (LC–MS/MS) Analysis

Plasma EVs from 3 mice in each group were isolated for proteomic analysis. The purified EVs were digested with trypsin (Promega, USA). Then the proteins were incubated with 5 mM dithiothreitol (Sigma, USA) for 45 min at 56°C for reduction, and alkylated with 20 mM iodoacetamide (Sigma) at room temperature for 15 min in the dark. Subsequently, the samples were resolved in 100 mM NH_4_HCO_3_ at a final concentration of 2 M. At last, proteins were digested at a 1:30 trypsin-to-protein mass ratio overnight at 37°C. After digestion, the peptides were processed by QExactive™ Plus (Thermo Scientific) coupled online to UPLC allowing peptide separation and identification. The electro spray voltage applied was 2.0 KV. The m/z scan range was 350 to 1,550 for full scan, and intact peptides were detected in the orbitrap at a resolution of 60,000. The scanning range of secondary mass spectrometry is fixed at 100 m/z and the resolution of secondary scanning is set to 15,000. The data acquisition mode uses a data-dependent scanning (DDA) program, in which the parent ions of the first 10 peptides with the highest signal intensity are selected to enter the HCD collision cell in turn and fragmented with 35% fragmentation energy, followed by secondary mass spectrometry. In order to improve the effective utilization of mass spectrometry, the automatic gain control (AGC) is set to 5e4, the signal threshold is set to 5,000 ions/s, the maximum injection time is set to 200 ms, and the dynamic exclusion time of tandem mass spectrometry is set to 30 s to avoid repeated scanning of parent ions.

### Database Search

The resulting LC–MS/MS data were processed using Maxquant search engine (v.1.5.2.8). Tandem mass spectra were searched against the “Mouse” database in UniProt and *E. granulosus* (28283 sequences) databases in the NCBI. An inverse library was added to calculate the false positive rate (FDR) caused by random matching, and a common contamination library was added to the database to eliminate the effect of contaminated proteins in the identification results. Set the restriction enzyme digestion method to trypsin/P, the number of missed cleavage sites is set to 2, the minimum length of peptide was set to 7 amino acid residues, and the maximum modification number of peptides was set to 5. The mass error tolerance of primary parent ion of first search and main search was set to 20 ppm and 0.02 Da of secondary fragment ion of 5 ppm, respectively. The FDR of protein identification was set to 1%. We determined the relative quantity of protein on the basis of the protein intensity among the different samples. The MS proteomics data have been deposited to the Proteome Xchange Consortium *via* the PRoteomics IDEntifications (PRIDE) partner repository with the dataset identifier PXD0025420.

### Proteomic Downstream Analysis

We annotated the identified proteins using the UniProt database (http://www.uniprot.org/). Complete proteomic data set from mice infected with plasma EVs at different stages was presented in **Supplementary Tables 1** and **2**. Additionally, we also functionally annotated the proteins, including gene ontology (GO) analysis (http://www.ebi.ac.uk/GOA/). In addition, we performed the REACTOME pathway analysis and PANTHER pathway analysis using the KOBAS database (http://kobas.cbi.pku.edu.cn/kobas3/). We used the WOLF PSORT software, an updated version of PSORT/PSORT II, for the prediction of eukaryotic sequences in order to predict subcellular localization. Moreover, we compared the identified proteins with those cataloged in the ExoCarta database (http://www.exocarta.org/).

EVs from HCF-EVs, PCS-EVs and the serum of CE were used for proteomic downstream. We analyzed the proteomic data of HCF and PCS-EVs from the MS proteomics data (PXDO14354) (Zhou et al., 2019) and the proteomic data of EVs from CE patient serum from our previous study (Wang W. et al., 2019). We compared the identified *E. granulosus*-derived proteins from EVs among HCF, PCS and the serum of CE.

### *In Vitro* Co-Culture of EVs and Spleen Mononuclear Cells

After the SPF normal mice without infection were euthanized, the spleens were ground with the plug of a 5 ml syringe to separate the mononuclear cells. Spleen mononuclear cells (5 × 10^5^ cells per well) were plated in 96-well flat-bottom culture plates (Corning-Costar, USA) with 250 μl of RPMI 1640 (Gibco, USA) containing 10% exosome-depleted fetal bovine serum (SBI) and 1% penicillin–streptomycin (Gibco) and incubated at 37°C and 5% carbon dioxide (CO_2_). The trypan blue staining was performed to observe the activity of spleen mononuclear cell. The following steps were carried out when the activity was more than 95%.

We added 5 μg of proteins of plasma EVs from mice at different stages of infection in a final volume of 50 μl of PBS to 5 × 10^5^ spleen mononuclear cells without any stimulation to observe the change in the number of B, CD4^+^ T, and CD8^+^ T cells, Treg cells, and MDSC, and added 50 μl of PBS and stimulator, anti-0.1 μg/ml CD3 (R&D Systems, USA) and anti-0.25 μg/ml CD28 (R&D Systems) antibodies, to the same culture system as two types of control. EVs and spleen mononuclear cells are incubated in at 37°C and 5% CO_2_ for 3 days. The supernatant was collected and frozen at −80°C. Cytokine levels were assayed by the Cytometric Bead Array Mouse Th1/Th2/Th17 Cytokine kit (BD Biosciences, USA) according to the protocol of the manufacturer.

### EV Labeling and Uptake by Host Cells

We labeled 5 μg of EVs with the green lipophilic fluorescent dye PKH-67 (Sigma-Aldrich, USA) as previously described with some modifications (Liu et al., 2018). Briefly, we transferred 5 μg of EVs to 12 ml of 1640 medium in a conical-bottom polypropylene tube. EVs were centrifuged at 110,000×*g* for 90 min at 4°C in a Sorvall WX+ Ultracentrifuge (Thermo Scientific) with rotor T-1270 and to precipitate EVs. Subsequently, the supernatant was carefully aspirated. We added 1 ml of Diluent C to the EV pellet to prepare a 2× EV suspension solution. We mixed 1 ml of Diluent C with 2 μl of PKH67 to prepare 2× Dye Solution. The both were rapidly mixed, and the mixture was incubated for 4 min at room temperature. The labeling reaction was stopped by adding an equal volume of 1% BSA to the labeling solution. Excess dye was removed by ultracentrifugation at 110,000×*g* for 90 min with RPMI 1640 medium. To verify the internalization of EVs by spleen immune cells, we labeled 5 μg of EVs from different groups (i.e., plasma, PCS, and HCF) with PKH-67 and added them to 5 × 10^5^ spleen mononuclear cells, which were incubated for 4 h at 37°C and 5% CO_2_. Afterward, we collected and washed the cells with a staining buffer (Biolegend, USA).

The strategy gates for immune cell analysis are shown in **Supplementary Figure 2**.

### Flow Cytometry and Imaging Flow Cytometer

Antibodies directed against the following makers were used to stain spleen mononuclear immune cells for flow cytometry analysis. Flow antibodies are shown in **Supplementary Table 3**. Approximately 5 μg of plasma EVs of infected mice at 0, 7, and 20 weeks were cultured with 5 × 10^5^ cells for 4 h at 37°C and 5% CO_2_. Approximately 5 μg of PCS and HCF-EVs co-cultured with spleen mononuclear cells for 4 h at 37°C and 5% CO_2_, respectively. We co-cultured the labeled PKH-67 EVs and spleen mononuclear cells at 4°C as a negative control.

For the uptake assays, 10,000 live-cells for each sample were acquired using a FlowSight Imaging Flow Cytometer (Amnis Corporation, USA) equipped with INSPIRE software and data were further analyzed using IDEAS software (Amnis Corporation) as previously described (Liu et al., 2018). Samples and single-color compensation controls were acquired at ×20 magnification. For the internalization percentage value, we calculated the percentage as a ratio of the number of cells internalizing EVs to the number of corresponding cell subpopulations.

### Statistical Analysis

Data are represented as the mean ± SD by GraphPad Prism 5 software for statistical analysis. Images were performed with Adobe Photoshop CS3.

For *in vitro* differentiation experiment and the detection of cytokine levels, one-way analysis of variance (ANOVA) for multi-group comparisons was performed among multiple groups. The *post-hoc* test is Tukey’s multiple comparisons test. Independent sample *t*-test was used for comparison between HCF-and PCS-EVs groups. For internalization experiment, one-way analysis of variance (ANOVA) for 0, 7, and 20 W-EVs groups were performed among multiple groups. The *post-hoc* test is Tukey’s multiple comparisons test. *P*-values were considered significant when *P <*0.05.

The differentially expressed proteins are performed by proteomic label-free quantitative analysis. Enrichment analysis of functional enrichment was performed by two-tailed Fisher’s exact test. The corrected *P*-value <0.05 was considered significant. The label free quantitative intensity of protein in each sample was obtained by non-standard quantitative calculation method, and the relative quantitative value of each sample was obtained according to the protein label free quantitative intensity between different samples. When *P*-value <0.05, the fold change of differentially expression was more than 1.5 as the significant upregulation threshold, and less than 1/1.5 as the significant downregulation threshold.

## Results

### Identification and Characterization of EVs

To verify the successful establishment of the *E. granulosus* infection mouse model, we examined the development of protoscoleces from hydatid cysts in the abdominal cavity of mice. No cysts of *E. granulosus* were seen in the mice at 7 weeks post-infection and uninfected mice. The typical structure of hydatid cysts had developed in mice at 20 weeks post-infection. Cysts had a mean diameter of 3.2 ± 1.5 mm and approximately five cysts were found in mice (**Supplementary Figure 1B**).

**Figure 1:**
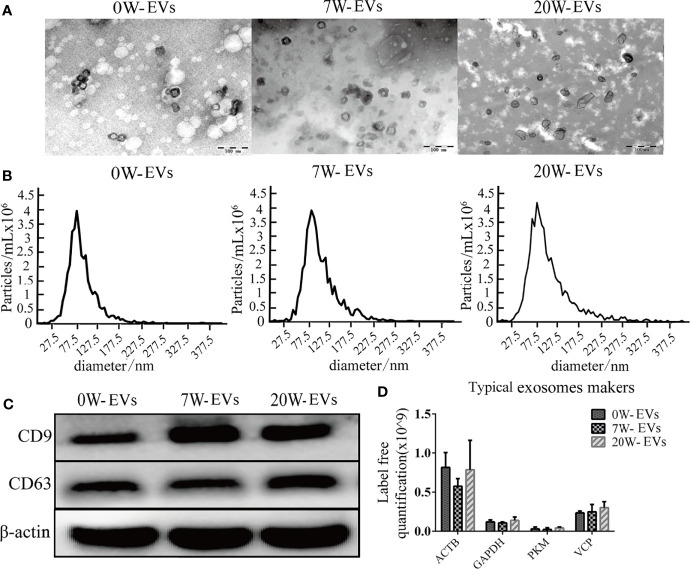
Identification and characterization of plasma EVs. **(A)** Transmission electron microscopy (TEM) images of plasma-EVs, Bar = 100 nm. **(B)** Size distribution of EVs was analyzed by nanoparticle tracking analysis (NTA). **(C)** Western blot analysis of CD9, CD63, and β-actin in EVs. **(D)** Typical exosome-like vesicles marker proteins Actin, cytoplasmic (ACTB), Glyceraldehyde-3-phosphate dehydrogenase (GAPDH), Pyruvate kinase PKM (PKM), Transitional endoplasmic reticulum ATPase (VCP) were identified in plasma EVs by mass spectrometry analyses.

To prove the successful isolation of plasma EVs from mice at different stages, we used TEM and NTA to evaluate the morphology and size distribution of the EVs. TEM suggested that EVs were round or cup-shaped and surrounded by a membrane bilayer with a diameter of approximately 100 nm (**Figure 1A**). NTA also indicated that the majority of EVs ranged from 30 to 150 nm in diameter (**Figure 1B**). The size, concentration and morphology of EVs from plasma of infected mice at different stages were similar. To further characterize EVs, we used western blot to reveal the expression of the typical exosome markers CD9 and CD63 (**Figure 1C**). In addition, we compared our data with exosome markers from the ExoCarta database. We found that ACTB, GAPDH, PKM and VCP were present in the EVs, as detected by mass spectrometry (**Figure 1D**). The concentration of per isolated EVs and their number of particles are shown in **Supplementary Table 4**. In addition, the isolated plasma EVs were not contaminated with endotoxin, that cannot interference cell cultural experiment (**Supplementary Figure 3**). These results indicated that our vesicles were EVs.

The characteristics of HCF and PCS-EVs were described by western blot for EVs maker of CD9 and CD63, TEM for morphology and NTA for size distribution as our previous research (Zhou et al., 2019).

### Comparison of the Proteins of EVs From Mouse Plasma at Different Stages of *E. granulosus* Infection

**Figure 2:**
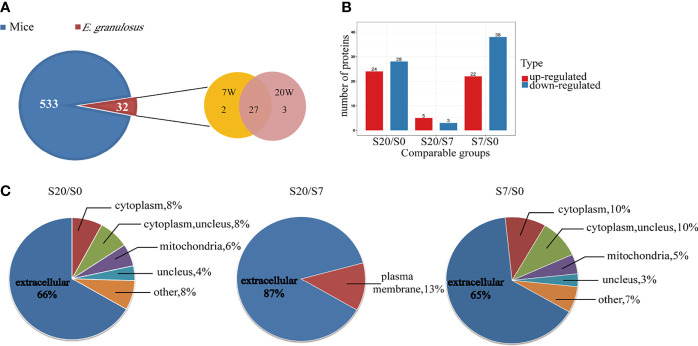
Proteomic analysis of differential proteins from plasma EVs of mice infected with *E. granulosus* at 0 week (0 W), 7 weeks (7 W), 20 weeks (20 W). **(A)** Venn diagram of protein components contained in plasma EVs from mice and *E. granulosus.* The blue area represents the mice proteins, and the red area represents the *E. granulosus* proteins, where in the orange area represents the proteins from 7 W-EVs and the pink area represents the proteins from 20 W-EVs. **(B)** Histogram analysis of different expression proteins among 0 W-, 7 W- and 20 W-EVs. S0, S7 and S20 are used to represent the signal intensity of 0 W-, 7 W- and 20 W-EVs detected by mass spectrometry, respectively. The red color represents upregulated proteins. The blue color represents downregulated proteins. A protein with a portion satisfying ratio more than 1.5 or less than 1/1.5, and a *P-*value <0.05 is regarded as a different protein. **(C)** The pie chart of subcellular localization of differentially expressed proteins among S20/S0, S20/S7, S7/S0.

To characterize the proteins and analysis the possible differences among EVs from infected mouse plasma at different stages. The distribution of peptide length identified and the quality precision of mass spectrometer met the requirements of quality control (**Supplementary Figure 4**). The proteomic analysis identified a total of 564 proteins; of these, 32 proteins matched the *E. granulosus* database, which we call *E. granulosus*-like proteins. Among these 32 proteins, 2 and 3 were specifically expressed in 7 W- and 20 W-EVs, respectively. In addition, 27 parasitic proteins were co-expressed in 7W- and 20 W-EVs (**Figure 2A**). The proteomic results revealed that 27 proteins were upregulated and 28 proteins were downregulated in 20 W-EVs compared with 0 W-EVs; 5 proteins were upregulated and 3 proteins were downregulated in 20 W-EVs compared with 7 W-EVs; and 22 proteins were upregulated and 38 proteins were downregulated in 7 W-EVs compared with 0 W-EVs (**Figure 2B** and **Supplementary Table 5**).

Moreover, we performed subcellular classification to explore the composition of differentially expressed EVs proteins. The results suggested that 66, 87, and 65% of differentially expressed proteins based on S20/S0, S20/S7, and S7/S0 were located in the extracellular space, respectively (**Figure 2C** and **Supplementary Table 6**).

**Figure 3:**
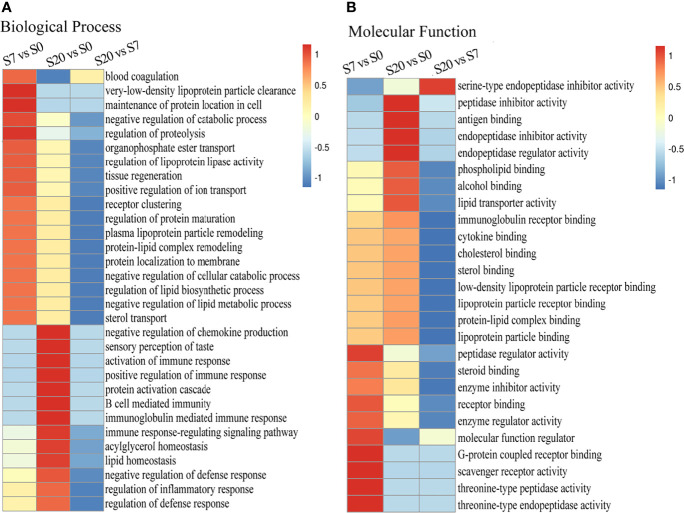
The Go enrichment of differentially expressed proteins among S20/S0, S20/S7, S7/S0 group. The heat map of functional enrichment study based on biological process **(A)** and molecular function **(B)** of differentially expression proteins presented in 0, 7, 20 W-EVs. We first collated all the categories obtained after enrichment along with their *P*-values, and then filtered for those categories which were at least enriched in one of the clusters with *P*-value <0.05. This filtered *P*-value matrix was transformed by the function x = −log10 (*P*-value). Finally, these x values were transformed for each functional category. These scores were then clustered by one-way hierarchical clustering in Genesis. Cluster membership was visualized by a heat map using the “heat map” function from the “gplots” R-package. The horizontal side of the heat map represents the results of the enrichment test for different groups, and the vertical side shows the differential expression of enrichment-related functions. The color blocks corresponding to the descriptions of differentially expressed proteins and functions in different groups indicate the degree of enrichment. Red represents a high degree of enrichment and blue represents a low degree of enrichment. Values −1 to 1 represented the degree of enrichment.

The GO functional enrichment was divided into two parts, namely, biological process and molecular function. On the one hand, the biological process showed that the protein functional enrichment of “blood coagulation, negative regulation of catabolic process, regulation of proteolysis” was high in differentially expressed proteins between 7 W- and 0 W-EVs. Then, the protein functional enrichment of “negative regulation of chemokines production, activation of immune response, positive regulation of immune response, B cell mediated immunity” was high in differential proteins in S20/S0 (**Figure 3A**). In short, the biological processes suggested that differentially expressed proteins of S7/S0 and S20/S0 are associated with the metabolism of proteins and immune response, respectively. On the other hand, the molecular function of “the antigen binding, peptidase inhibitor activity, cytokine binding” was enriched in differential protein compared 20 W and 0 W-EVs, and “receptor binding, molecular function regulator” was highly enriched in differential proteins of S7/S0 (**Figure 3B**). The above results both indicated the differentially expressed proteins of EVs have the important role in biological process and molecular function.

**Figure 4:**
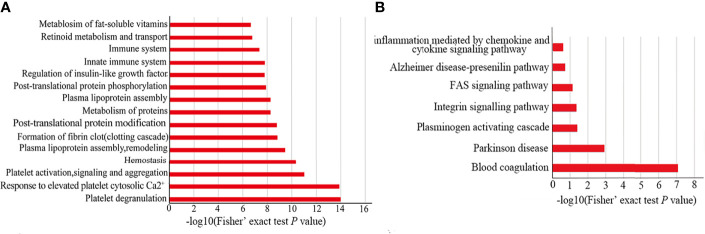
Functional enrichment for differentially expressed proteins. The bar chart of REACTOME **(A)** and PANTHER **(B)** pathway enrichment for all differentially expressed proteins.

In order to explore the possible functions of all different proteins, we performed REACTOME and PANTHER pathway analysis. From the REACTOME pathway enrichment analysis, the pathways of “platelet degranulation, response to elevated platelet cytosolic Ca^+^ and platelet activation, signaling and aggregation” are the 3 top pathways (**Figure 4A**). Furthermore, there are two important pathways related to the processes of the host against parasites (i.e., innate immune and immune system) (**Figure 4A**). The results of our PANTHER pathway enrichment analysis also revealed blood coagulation with most enrichment (**Figure 4B**). These results indicated that differentially expressed proteins are associated with platelet and blood coagulation, which may be related to the EVs both came from plasma. Furthermore, the results further indicated that the proteins of EVs played an important role in participating in the host immune response.

The data was further analyzed from different points of view such as comparing with proteomic data from parasite-derived EVs (namely, HCF-EVs, PCS-EVs) and host-derived EVs from the serum of CE patients (**Table 1**). The above implied these EVs provide several important *E. granulosus* infection-related proteins.

**Table 1:** Comparison of *E. granulosus* derived-proteins among EVs from serum of CE patient, mouse plasma after 7, 20 W infection, hydatid cyst fluid (HCF), and protoscolex culture supernatant (PCS).

Order	Protein accession	Protein description	HCF-EVs	PCS-EVs	CE serum-EVs	Expressed stages
1	O95810	Caveolase-associated protein 2			√	20 W
2	P02788	Lactotransferrin			√	7, 20 W
3	P05106	Integrin beta-3			√	7, 20 W
4	XP_024345893.1	Heat shock cognate protein	√	√		7 W
5	XP_024354730.1	Rab (Ras-related protein Rap-1b)	√	√		20 W
7	XP_024347275.1	Actin-1	√	√		7, 20 W
6	XP_024346672.1	Heat shock cognate protein	√			7 W
8	XP_024351663.1	Glutathione S-transferase class-mu isozyme	√			20 W
9	XP_024348354.1	Katanin p60 ATPase-containing subunit A-like 2	√			7, 20 W
10	XP_024351059.1	Glutaminyl-peptide cyclotransferase-like protein	√			7, 20 W
11	XP_024354458.1	26S protease regulatory subunit 6B	√			7, 20 W
12	XP_024355946.1	Low-density lipoprotein receptor-related protein	√			7, 20 W

The data of proteins of EVs from HCF, PCS and serum of CE patient from previous study (Wang et al., 2019; Zhou et al., 2019).

### EVs Are Efficiently Taken up by Spleen Mononuclear Cells

**Figure 5:**
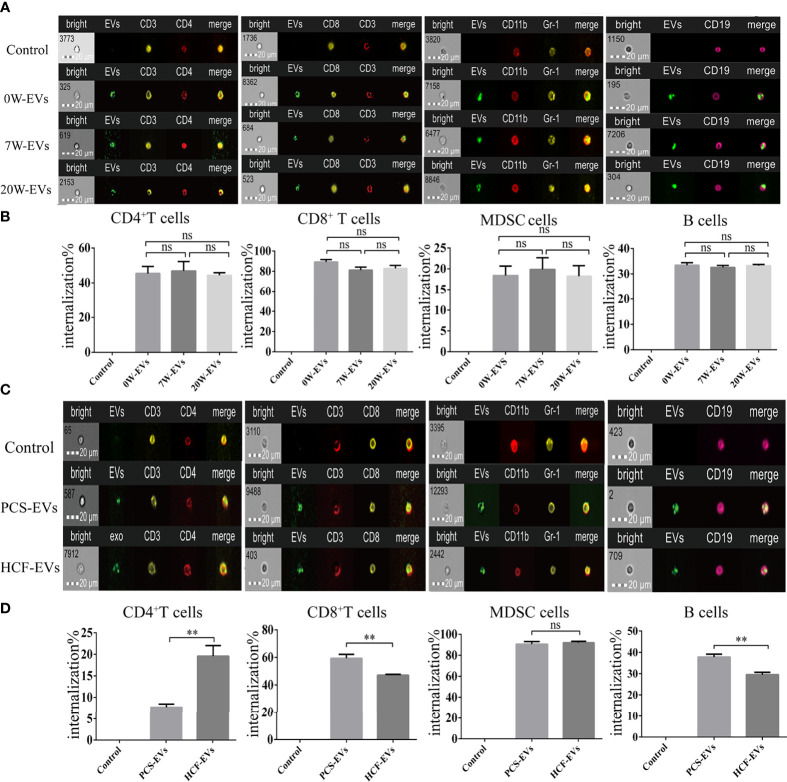
EVs are efficiently taken up by spleen mononuclear cells. Approximately 5 μg of EVs labeled with PKH-67 were incubated with 5 × 10^5^ spleen mononuclear cells for 4 h at 37°C and 5% CO_2_, while at 4°C as a negative control. The cells were then collected for detecting the uptake percentage of EVs by different types of cells, namely, B cells, MDSC, CD4^+^ and CD8^+^ T cells. Scale bars represent 20 μm. **(A)** The image of flow cytometers and **(B)** bar graph for internalization percentage of 0, 7, and 20 W-plasma EVs (PKH67-green) by CD4^+^ T cells (CD3^+^ labeled by PE-yellow and CD4^+^ labeled by Percp-red), CD8^+^ T cells (CD3^+^ labeled by Percp Cy5.5-red and CD8^+^ labeled by PE-yellow), MDSC (CD11b labeled by PE-Cy5-red and Gr-1 labeled by PE-red), B cells (CD19 labeled by PE-Cy7-pink). Comparisons among groups were performed using one-way ANOVA (*post-hoc* test is Tukey’s multiple comparisons test), ns, not significant. **P < 0.01. **(C)** The image of flow cytometers and **(D)** bar graph for internalization percentage of PCS and HCF-EVs (PKH67-green) by CD4^+^ T cells (CD3^+^ labeled by PE-yellow, CD4^+^ labeled by Percp-red), CD8^+^ T cells (CD3^+^ labeled by Percp Cy5.5-red, CD8^+^ labeled by PE-yellow), MDSC (CD11b labeled by PE-Cy5-red, Gr-1 labeled by PE-red), and B cells (CD19 labeled by PE-Cy7-pink). The independent sample *t*-test was used to compare the HCF- and PCS-EVs groups. Data were presented as the mean ± SD of at least three independent experiments. **P < 0.01, ns, not significant.

Since internalization of EVs is one of the main mechanisms of cargo delivery to recipient cells, we explored the internalization of EVs by immune cells. As shown by spot count and fluorescence intensity, plasma EVs were internalized into the immune cells. The results show that degree of internalization of plasma EVs from mice at different infection stages were similar in the same immune cells, namely, CD4^+^ and CD8^+^ T cells, B cells and MDSC (**Figures 5A, B**), which may be related to the origin of EVs and the mechanism of internalization. The *E. granulosus* includes two important conditions, protoscoleces, and hydatid cysts. We further evaluated the degree of PCS-EVs and HCF-EVs were taken up by spleen mononuclear cells. The degree of internalization of PCS-EVs was higher than HCF-EVs by B cells and CD8^+^ T cells, and the degree of internalization of HCF-EVs were higher than PCS-EVs by CD4^+^ T cells. The uptake of PCS-EVs and HCF-EVs were similar in MDSC (**Figures 5C, D**).

### EVs Can Influence the Differentiation of Immune Cells

**Figure 6:**
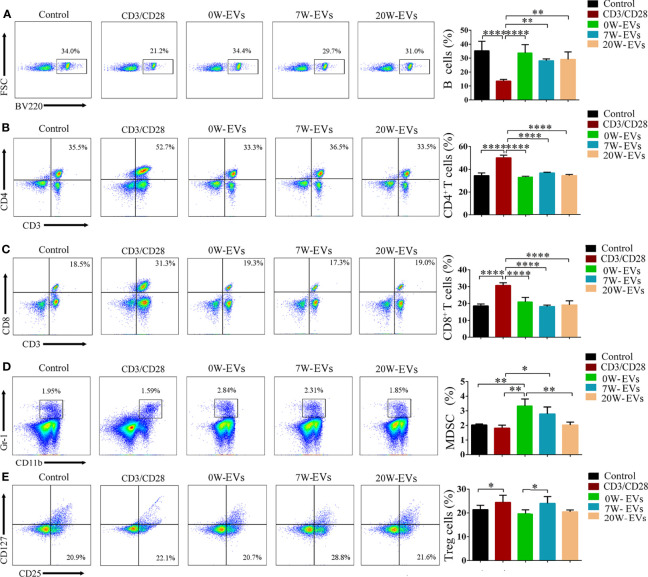
EVs can affect the differentiation of immune cells. Plasma EVs, isolated from infected mice at different stages, namely, uninfected (0 W), 7-week post-infection (7 W), 20-week post-infection (20 W), co-cultured with spleen mononuclear cells for 5 days without other stimulation. PBS co-cultured with spleen mononuclear cells without stimulation as control, with stimulation of anti-CD3 and anti-CD28 antibody as another control. The effect of EVs on the number of **(A)** B cells, **(B)** CD4^+^ T cells, **(C)** CD8^+^ T cells, **(D)** MDSC, and **(E)** Treg cells. Treg cells were shown from CD3^+^ (labeled by APC) and CD4^+^ (labeled by FITC) cells. A representative experiment is shown. Data were presented as the mean ± SD of at least three independent experiments (n = 5). Comparisons among groups were performed with one-way ANOVA. *P < 0.05, **P < 0.01, ****P < 0.0001.

To observe the effect of EVs on immune cells, we incubated plasma EVs from infected mice at different stages with immune cells. The results suggested that plasma EVs from different infection stages had no significant effect on the differentiation of B cells and CD4^+^ T and CD8^+^ T cells (**Figures 6A**–**C**). Importantly, 20 W-EVs and 7 W-EVs can decrease the related abundant of MDSC compared to 0 W-EVs (**Figure 6D**), 7 W-EVs can increase the number of Treg cells compared to 0 W- and 20 W-EVs, whereas there was no significant difference between 0 W-EVs and 2 0W-EVs (**Figure 6E**). These results showed that the plasma EVs of mice infected at different stages had different effects on different immune cells.

### EVs Have Immunomodulatory Functions

**Figure 7:**
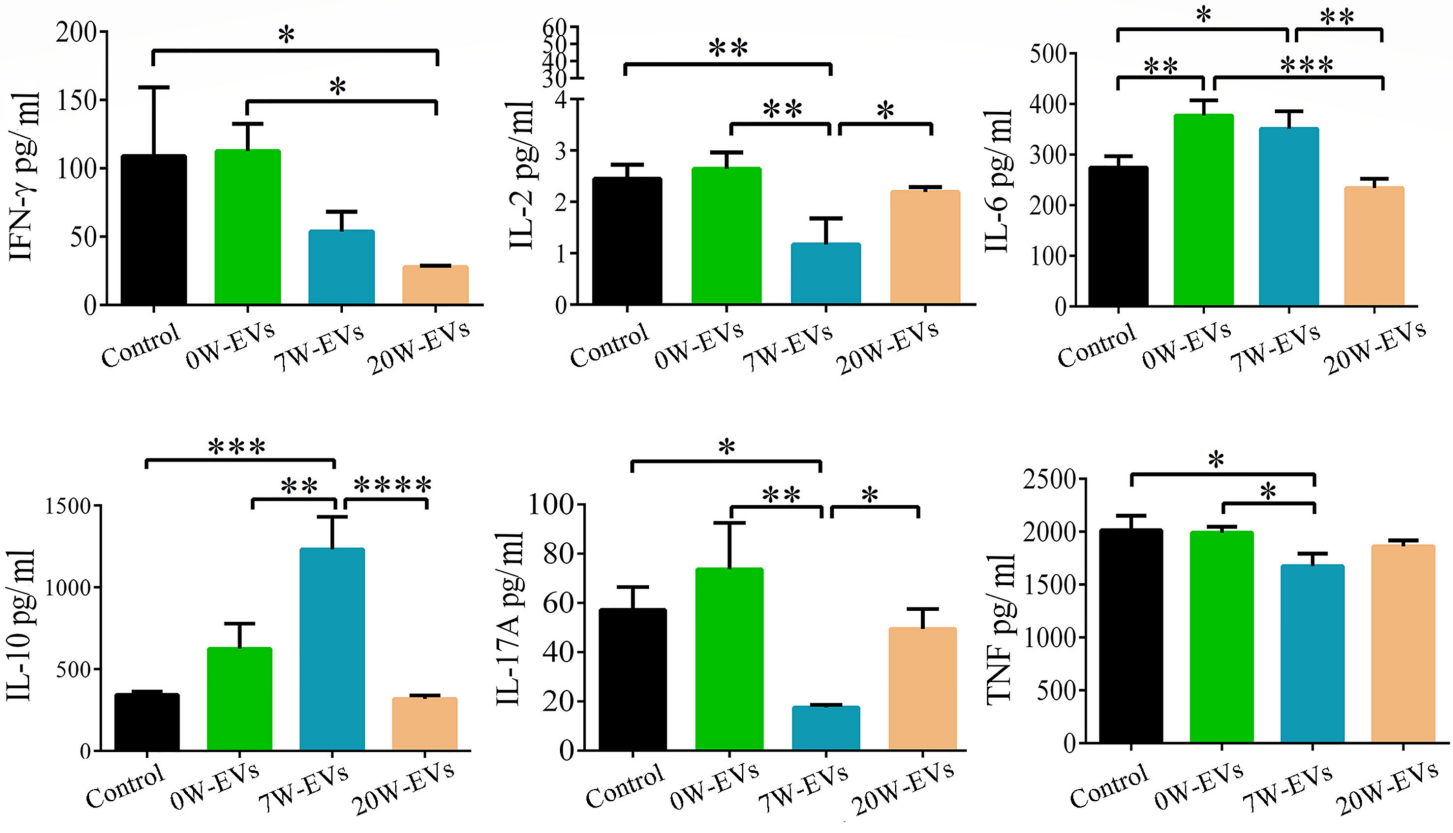
The effect of plasma EVs isolated from mice infected with *E. granulosus*at different stages on the secretion of cytokines in spleen mononuclear cells. The effect of plasma EVs co-cultured with spleen mononuclear cells for 5 days at 37°C and 5% CO_2_ without stimulation. PBS co-cultured with spleen mononuclear cells without stimulation as control, with stimulation of anti-CD3 and anti-CD28 antibodies as stimulation group. The levels of cytokines in the culture supernatant were detected by cytometric bead array Mouse Th1/Th2/Th17 Cytokine Kit. Comparisons among groups were performed with one-way ANOVA; *P < 0.05, **P < 0.01, ***P < 0.001, ****P < 0.0001. Data are mean ± S.D. of at least three independent experiments (n = 5).

To elucidate the functions of EVs in the immune response, we collected the co-culture supernatant to examine the levels of cytokines. We found that 20 W-EVs significantly reduced the levels of IFN-γ and IL-6 compared with 0 W-EVs. However, 7 W-EVs inhibited the secretion of IL-17A, TNF (total TNF), and IL-2, while promoting the secretion of IL-10 compared with 0 W-EVs. Compared with 7 W-EVs, the levels of IL-6 and IL-10 were decreased, and the levels of IL-17A and IL-2 were significantly increased by 20 W-EVs (**Figure 7**). An interesting phenomenon was observed that the regulation of 7 W- and 20 W-EVs on the level of cytokines secreted by immune cells was complex. Anti-CD3/CD28 antibodies can increase the level of total cytokine compared with control (**Supplementary Figure 5**), which indicated the immune cells were active. These results suggested that EVs played an immune regulation role.

## Discussion

Recent pieces of evidence have indicated that EVs played a certain role in communication involving in the processes of infection and persistence between parasites and host, such as intercellular communication and modulation of immune responses (Coakley et al., 2015). In the present study, we focused on proteomic analysis of the EVs circulating in the plasma of mouse during different infection stages of *E. granulosus* protoscoleces, especially before and after the formation of cysts. Subsequently, we analyzed protein functional enrichment and explored the effect of immune response though co-cultural experiments. Our results provided several important *E. granulosus* infection-related proteins in the process of CE, and further revealed that host-derived EVs played an important role in participating in communication and substance exchange between parasite and host. We further provided information for the understanding of the immune regulation of host-derived EVs between *E. granulosus* and host.

The results of our TEM (**Figure 1A**), NTA (**Figure 1B**), and western blot (**Figure 1C**) revealed that we succeeded to isolate EVs from plasma in mice infected with *E. granulosus*. Our proteomic data indicated that plasma EVs included characteristic markers of exosomes, such as Actin, cytoplasmic (ACTB), Glyceraldehyde-3-phosphate dehydrogenase (GAPDH), Pyruvate kinase PKM (PKM), Transitional endoplasmic reticulum ATPase (VCP) were identified in plasma EVs (**Figure 1D**). These results were consistent with the guidelines of the International Society for EVs 2018 (MISEV2018) (Thery et al., 2018). In addition, the results of subcellular localization in our proteomic analysis showed that most differentially expressed proteins were presented in the extracellular space, which can be explained by the generation through inward budding of the endosomal membrane of the EVs-like vesicles (Robbins and Morelli, 2014). Most of the extracellular proteins were enveloped by EVs, although there were some contaminating proteins, such as nuclear proteins and mitochondrial proteins. However, no methods are available to completely remove the contaminating proteins. But it still indicated that the purity of EVs was very high.

In the present study, our proteomic analysis of plasma EVs identified 533 and 32 proteins of mouse and *E. granulosus*-like proteins, respectively (**Supplementary Tables 1, 2**). Our results confirmed that plasma EVs played a role in the transmission of signaling and substances between parasite and host. Federica et al. also found some proteins in plasma exosomes from cystic echinococcosis patients that were also simultaneously presented in the HCF-EVs and PCS-EVs. Meanwhile, in our study, we also observed that the Rab (Ras-related protein Rap-1b) and actin proteins were identified in plasma EVs from mouse infected with *E. granulosus* protocoleces. The previous study suggested downregulation of Ras-related protein Rap-1b could effectively inhibit the tumorigenesis of thyroid cancer cells (Wang P. et al., 2019). Other researches proved that *E. granulosus* protein may provide a new prospect for cancer therapy (Guan et al., 2019). On the other hand, the results showed EVs can provide important *E. granulosus*-related proteins. If *E. granulosus*-related proteins were validated on a larger cohort, this would be a valuable improvement for CE diagnosis and management. Therefore, we speculate EVs secreted by *E. granulosus* protoscoleces can participate in the metabolism of the host and enter the blood plasma of the circulatory system, thus we can identify the same proteins of PCS-EVs (**Table 1**).

Previous reports have shown the changes in the status of immune cells after *E. granulosus* protoscolex infection. Some studies focused on early infection (Mourglia-Ettlin et al., 2011; Yin et al., 2017), revealing the changes of cytokines and immune cells on post-infection days 3, 5, 7, and 9 (Mourglia-Ettlin et al., 2016). Moreover, studies have shown the systemic immune status of innate and adaptive immunity at 30-, 180-, and 360-days post-infection to explore the interactions between host and parasite especially during persistent infection (Pan et al., 2013). As mentioned in studies, in the early stages of CE, a Th1 immune response dominates to protect from parasite infection by secreted IL-2, IFN-γ, TNF and so on. But during cyst establishment and growth, there is a switch to a Th2 response by secreted IL-4, IL-5, IL-6, IL-10 and so on, which is beneficial to parasite survival. Above the research results revealed that dynamic changes of cytokines in host immune response process during *E. granulosus* protoscolces infection process. In the present study, we firstly explored to the immune regulated function of plasma EVs from mice infected with *E. granulosus* protoscolces at different stages. Our results suggested that the changes of cytokine levels by secreted Th1/Th2/Th17 cells presented the complex phenomenon in co-cultured 0, 7, 20 W-EVs with immune cells. The previously reported that the percentage of Treg and MDSC cells were increased in mice in peripheral blood mononuclear cells (Coakley et al., 2017). This similar phenomenon was also observed in the present study. Co-cultural experiments have shown that 7W-EVs upregulated the relative abundance of Tregs (**Figure 6E**) and increased the expression of IL-10, decreased other cytokines secreted by Th cells compared to 0 W-EVs (**Figure 7**).

Our results are in agreement with other pieces of literature, where they also found that the excretory/secretory products of the *Echinococcus multilocularis* can induce Treg expression and IL-10 production by host T-cells *in vitro* (Nono et al., 2020). Interestingly, there were more unique peptides of Peptidyl-prolyl *cis-trans* isomerase A (PPIA) expressed in the 7 W-EVs compared to 0 and 20W-EVs (**Supplementary Table 1**). Previous research has reported that PPIA can modulate dendritic cell function in the induction of CD4^+^ T cell proliferation with a preferential expansion of Treg cells in *Shistosoma mansoni* worm secretome. Both *S. mansoni* and *E. granulosus* belong to the helminths, and PPIA may have played a similar function in host immune system, but the specific function of PPIA needs to be further studied in *E. granulosus* infection. Thus, we only speculate that the elevated number of Treg cells by 7 W-EVs may be related to the PPIA in 7 W-EVs. Nahid Ali et al. provided pieces of evidence of Treg cells as a source of IL-10 with parasite load during active disease (Bhattacharya et al., 2016). But for the whole spleen cells we cultured in this experiment, we have insufficient data to support that the increased IL-10 is secreted by Tregs. We just speculated that 7 W-EVs mainly exerted negative regulatory effects on immune cells, which may be related to the increase level of Treg cells and IL-10. This result is related to S7/S0 differential protein functional enrichment (**Figure 3A**), which both showed that EVs in plasma of early infected mice may have played an immune regulation function. Besides, lactotransferrin (or lactoferrin, Ltf) and integrin beta-3 (Itgb3) expressed in 7 W-EVs and 20 W-EVs (**Table 1**), and the ratio of S20/S7 of Ltf and Itgb3 is 1.274 and 1.36 respectively (**Supplementary Table 1**), both are more than 1, which means that the expression of these two proteins in 20 W-EVs is higher than that in 7 W-EVs. According to the relevant literature, Ltf is potential to reduce cytokine storm induced by SARS-CoV-2 (Zimecki et al., 2021). Itgb3 impairs recruitment of effector cells and alters cytokine production to prolong allograft survival, which can be used as a new target for immunosuppressive therapy (Lacy-Hulbert et al., 2007). Thus, both Ltf and Itgb3 have the ability to reduce cytokine levels. Overall, cytokine levels were lower in 7 W-EVs and 20 W-EVs than in 0 W-EVs, which may be related to the fact that Ltf and Itgb3 were not expressed in 0 W-EVs, and not detected in 0 W-EVs (**Figure 7** and **Table 1**). For 20 W-EVs, lower expression of IL-6 and IL-10 may be associated with higher levels of Ltf and Itgb3 compared to 7 W-EVs (**Figure 7** and **Table 1**).

Meanwhile, bioinformatics analyses of biological process indicated that differentially expressed proteins of S20/S0 could be involved in the negative regulation of chemokine production in the biological process (**Figure 3A**). Meanwhile, the results both further implied that the differential proteins of S20/S0 have a negative immune regulation function. This may be due to an immune response to the parasite by the infected host. This provides some basis for the regulatory role of host plasma EVs in the late stages of infection. However, the mechanisms of function of the special proteins in EVs need to be further investigated. Other than that, this finding also observed that the EVs played a multidirectional immune regulation role. This result may be explained by the fact that the components of EVs isolated from the plasma of circulatory system were complex, because the antigenic components that produce an immune response to the host are complex at the late infected stage. In addition, the effects of different EVs population on immune cells is potentially a result of EV-contained nucleic acids (e.g., small interfering miRNAs) (Sotillo et al., 2020; Zhang et al., 2020) and the proteomic cargo of the vesicles (Wu et al., 2018) during early and late stage of an infection. In this previous study, miRNA-4989 of *E. multilocularis* EVs have immunomodulatory effects (Ding et al., 2019). It is interesting to explore the reason behind this phenomenon, but the specific mechanism remains to be further research. These finding might help others to understand the effects of plasma EVs of infected mice during the development of CE on the immune response.

The results of REACTOME and PANTHER pathway enrichment showed that the different expressed proteins are high enriched in blood coagulation and platelet degranulation. It is reasonable that EVs are from plasma and it contains blood and platelet protein, so it is easier to enrich into blood coagulation, which also indicates that this process may be related to helminths infection (Yang et al., 2015).

The previous studies have already shown that helminth EVs can be internalized by macrophages by confocal microscopy (de la Torre-Escudero et al., 2019) and imaging flow cytometers (Coakley et al., 2017). In the present study, we further visualized that EVs were taken up simultaneously by different immune cells such as B cells, CD4^+^ and CD8^+^ T cells, and MDSC. The results from our internalization experiments revealed that no significant difference in the uptake degree of the immune cells treated with 0 W-, 7 W-, and 20 W-EVs from mouse plasma (**Figures 5A, B**). Related literature shows the way in which EVs are taken up by cells with a high degree of uptake with longer co-culture time (Franzen et al., 2014; Coakley et al., 2017). We compared the difference between 4 and 12 h of internalization of EVs by immune cells, but there was no difference (data not shown), which is consistent with the results of internalization of EVs secreted by *S. japonicum* (Liu et al., 2019). The results may be explained by the fact that immune cells have the same mechanisms to take up plasma EVs (Christianson et al., 2013; Pegtel and Gould, 2019). Moreover, our data further showed that EVs from PCS and HCF were internalized in CD4^+^ T cells, CD8^+^ T cells, B cells, and MDSC. The degree of internalization of PCS-EVs and HCF-EVs was different in the same type of immune cells, except for MDSC (**Figures 5C, D**). The previous study has already shown that PCS-EVs can be internalized dendritic cells. In this study we further supplemented that PCS-EVs can be taken up by different immune cells (Nicolao et al., 2019). In addition, it is known from the literature that the excretory–secretory products of *E. granulosus* protoscoleces directly regulated the differentiation of B10 and B17 cells (Pan et al., 2017), and our study further supplemented that PCS-EVs can be taken up by B cell. But whether EVs can regulate the function of B cells by internalization remains to be further studied. In addition, HCF-EVs contains complex components, namely, host, protoscoleces and cells of the germinal layer from cyst wall, which have different regulatory effects on immune response. Thus, it is reasonable that the ability of immune cells to uptake EVs from PCS and HCF was different. Thus, we research further highlighted the role of EVs involving in communication between parasite and host.

So far, it has been proposed that the cells internalize EVs either by fusion with the plasma membrane or *via* endocytosis (Mulcahy et al., 2014). Uptake *via* endocytosis can be categorized into the different types of endocytotic processes, namely, clathrin-mediated endocytosis, caveolin-mediated endocytosis, lipid raft-mediated endocytosis, macropinocytosis, and phagocytosis (Mulcahy et al., 2014). For example, Adrian et al. certificated that the uptake of exosomes decreased at 4°C compared to dendritic cells internalized PKH67-labeled exosomes within 2 h at 37°C *in vitro*, suggesting that exosomes were internalized actively rather than attached to the dendritic cell surface (Morelli et al., 2004). This study is consistent with our results.

In addition, researchers have already demonstrated that uptake by host cells *of T. vaginalis*-EVs depends on proteoglycans and caveolin-1, and their internalization proceeds is conducted by clathrin-independent lipid raft-mediated endocytosis (Rai and Johnson, 2019). Thus, there are different pathways for internalization of EVs in cells. In this study, the mechanism of EVs uptake by immune cells needs to be further investigated.

Because of the unique physical and biological properties of EVs, namely, high biocompatibility and intrinsic targeting activity, EVs have been intensively studied for their use as therapeutics (Gyorgy et al., 2015). EVs can be internalized by host cells and modulate the host immune response (Montaner et al., 2014). These EVs are also involved in the pathology of parasitic disease and have great potential to be a new diagnostic tools and therapeutic agents against different parasitic pathogens (Marcilla et al., 2014). In our study, we further identified that plasma-derived EVs from mouse infected with *E. granulosus* can be taken up by immune cells, but the effect of internalization on the pathological process of *E. granulosus* infection requires further study.

In summary, the current study focused on the proteomic analysis of the differential proteins of EVs from host plasma to explore the functional enrichment, and their effect on immune response during different stages of *E. granulosus* infection. The finding clearly indicated that EVs can be taken up by immune cells, and effected the levels of immune cells and cytokines secreted by immune cells. The vast majority of EVs used in the immunomodulation experiments are of mouse origin, the observed effects are more likely due to the host immune response to parasite infection. The finding contributed to understanding the function of infected host-derived EVs. The key strengths of this study were that differential proteins may be emphasis the immune regulation function of EVs from host infected with *E. granulosus* at different stages. While further research is required for the identification and function for *E. granulosus* infection-related proteins, at present, our team cannot perform further exploration own to limitation of the decrease in the number of CE patients. The mechanism of EVs to regulate host immune response needs to be further studied. Regardless of its limitations, the study certainly adds to our understanding of the importance of EVs, which supplemented unique dataset for this *E. granulosus.*

## Data Availability Statement

The datasets presented in this study can be found in online repositories. The names of the repository/repositories and accession number(s) can be found in the article/**Supplementary Material**.

## Ethics Statement

The studies involving human participants were reviewed and approved by the Ningxia Medical University Medical Ethical Committee (permit number: 2019-62). The patients/participants provided their written informed consent to participate in this study. The animal study was reviewed and approved by the Ningxia Medical University Medical Ethical Committee(permit number: 2021-846).

## Author Contributions

Conceptualization: JZ, SD. Investigation: CS, HY. Methodology: CS, XZ, XG. Formal analysis: XZ, WY. Supervision: JZ, SD. Visualization: JW, MB, YZ, MY. Writing-original draft: CS, XZ. Writing-review & editing: AN, WZ, JZ. All authors listed have made a substantial, direct, and intellectual contribution to the work and approved it for publication.

## Funding

This work was supported by the National Natural Science Foundation of China (32160181), the Ningxia Natural Science Found Project (2020AAC03426), the Ningxia Medical University Scientific Research Project (XY201526), and the Ningxia High School first-class Disciplines (West China first-class Disciplines Basic Medical Sc iences at Ningxia Medical University;NXYLXK2017B07). The funders had no role in study design,data collection and analysis, decision to publish, or preparationof the manuscript.

## Conflict of Interest

The authors declare that the research was conducted in the absence of any commercial or financial relationships that could be construed as a potential conflict of interest.

## Publisher’s Note

All claims expressed in this article are solely those of the authors and do not necessarily represent those of their affiliated organizations, or those of the publisher, the editors and the reviewers. Any product that may be evaluated in this article, or claim that may be made by its manufacturer, is not guaranteed or endorsed by the publisher.
