# Supplementary material for: Proteomic Analysis of Plasma-Derived Extracellular Vesicles From Mice With Echinococcus granulosus at Different Infection Stages and Their Immunomodulatory Functions
Source: Front Cell Infect Microbiol. 2022 Mar 10;12:805010. doi: 10.3389/fcimb.2022.805010 (PMC8960237; doi:10.3389/fcimb.2022.805010)
Supplement: Supplementary file 1 [file DataSheet_1.docx]

Supplementary Material

## Supplementary Figures

##
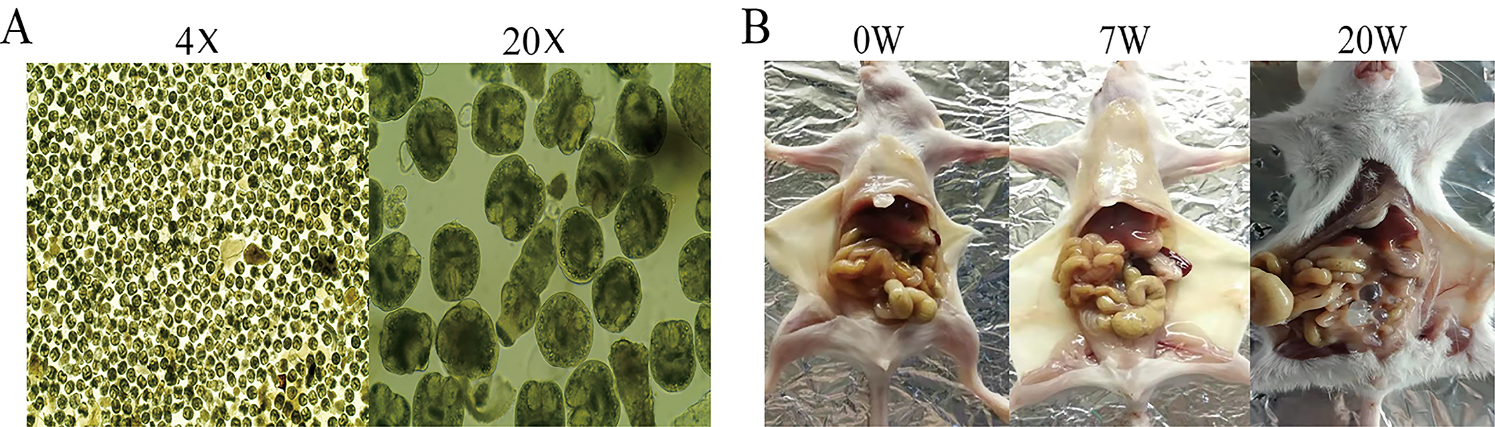


**Supplementary Figure 1. (A) Structural integrity of protoscoleces were observed under microscope with magnification 4 times (4×) and 20 times (20×), respectively. (B) Development of protoscoleces in mice at different stages of infection.**


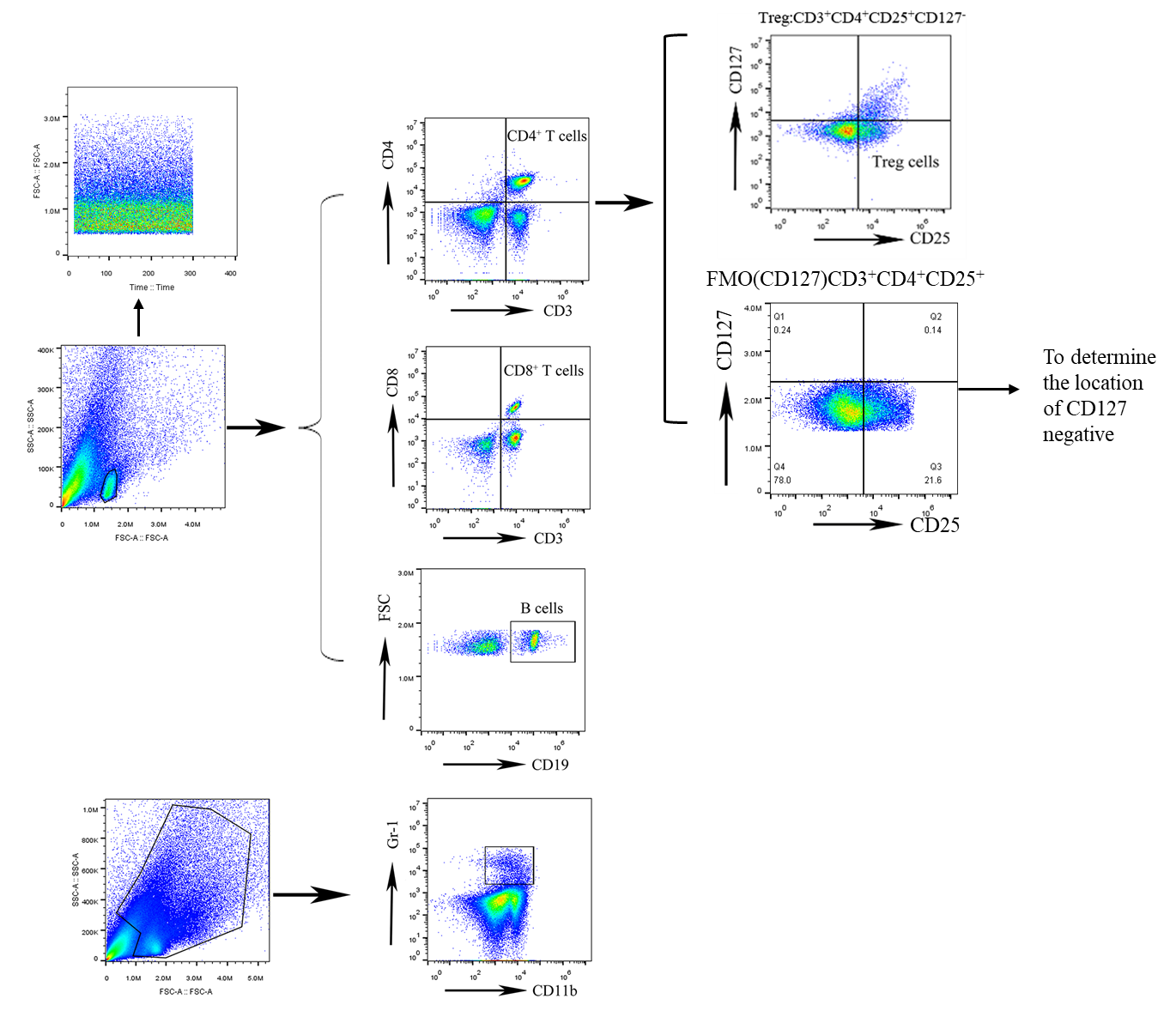


**Supplementary Figure 2. The strategy gates for immune cell analysis were descripted.CD4^+^ T cell, CD8^+^ T cells, B cells were targeted in lymphocytes. Treg cells were targeted in CD3^+^ and CD4^+^ cells. MDSC were targeted in granulocyte.**

##
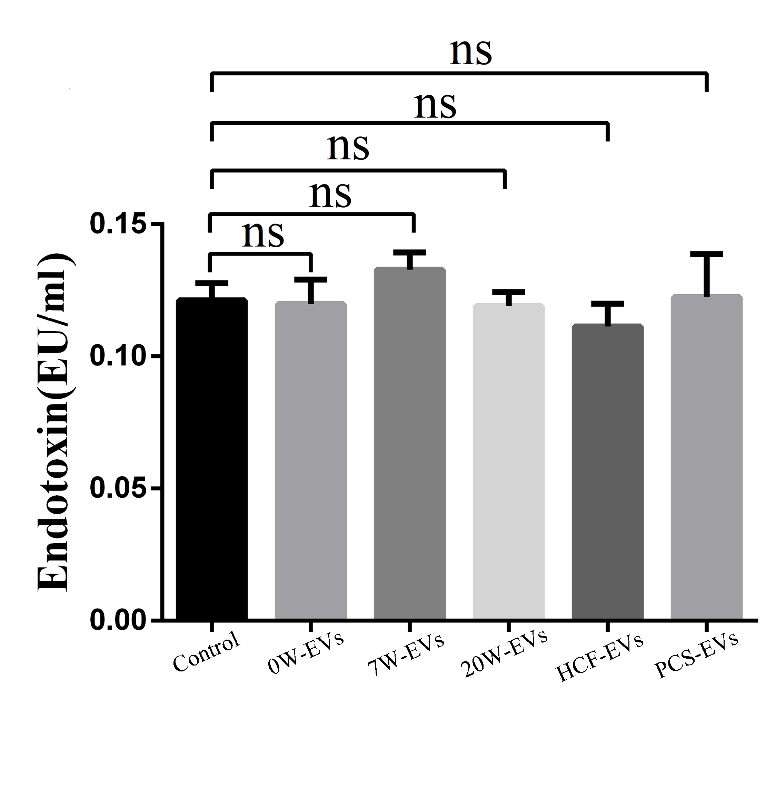


**Supplementary Figure 3. The level of endotoxin in 0-, 7-, 20W-EVs and HCF and PCS-EVs. Student *t* test was used for comparison between the two groups. ns, not significant.**

**
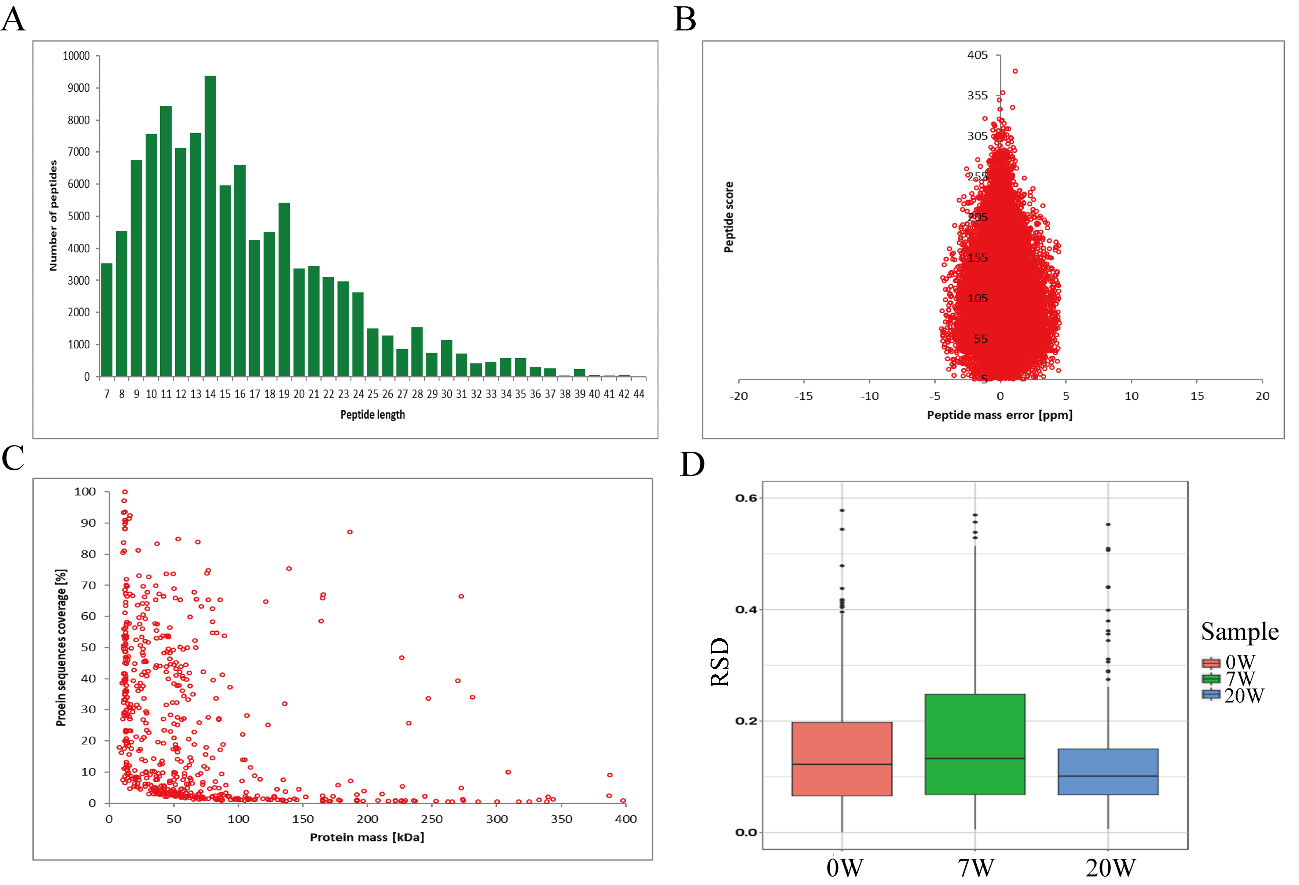
**

**Supplementary Figure 4. The distribution of peptide length identified and the quality precision of mass spectrometer meeting the requirements of quality control. (A) Length distribution of peptide identified by mass spectrometry. (B) Mass precision distribution of mass spectrometer. (C) Relationship between protein molecular weight and coverage identified by mass spectrometry. (D) Boxplot of quantitative RSD distribution of protein between repeated samples.**

**
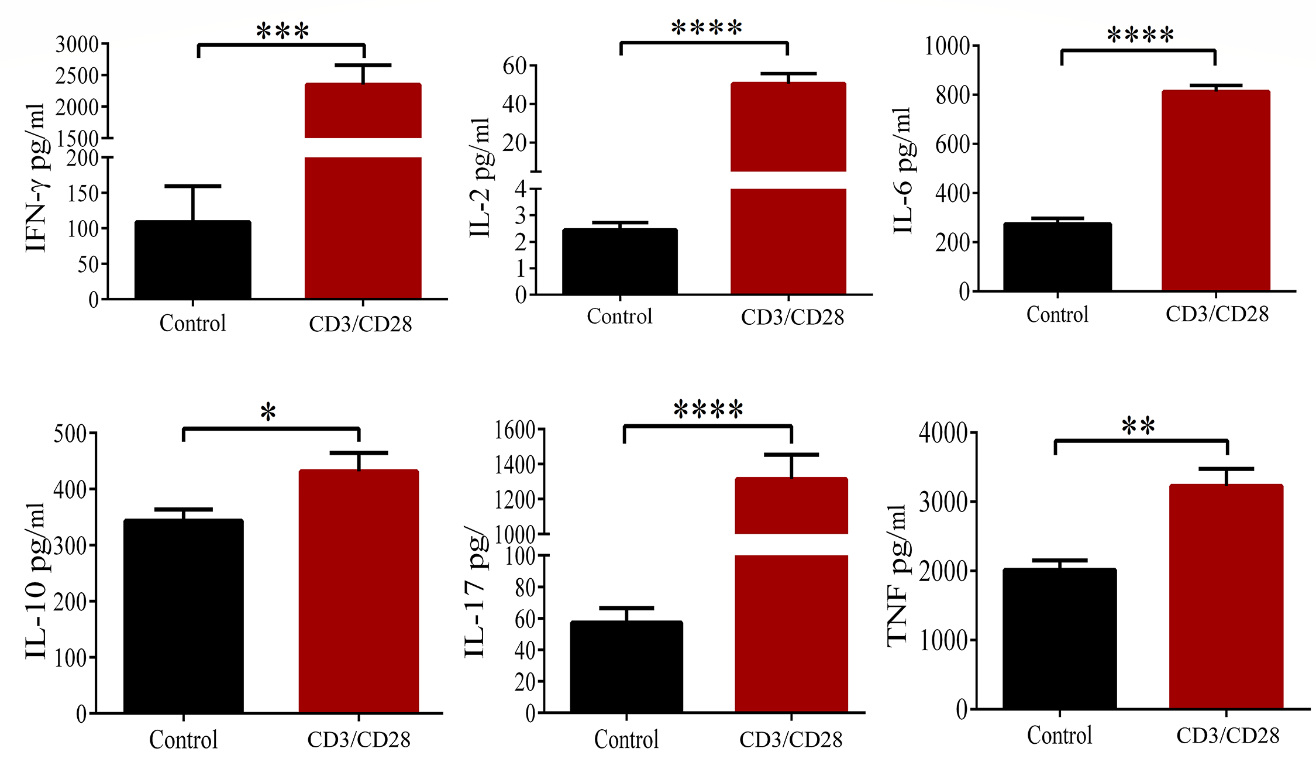
**

**Supplementary Figure 5. The levels of cytokines were compared between control and CD3/CD28 groups. PBS and spleen cells were co-cultured as control group. CD3/CD28 antibodies and spleen cells were co-cultured as CD3/CD28 group. Independent sample *t* test was used for comparison between the two groups. **P*<0.05, ***P<*0.01, ****P*<0.001, *****P*<0.0001.**
