# Supplementary material for: Proteomic Analysis of Plasma-Derived Extracellular Vesicles From Mice With Echinococcus granulosus at Different Infection Stages and Their Immunomodulatory Functions
Source: Front Cell Infect Microbiol. 2022 Mar 10;12:805010. doi: 10.3389/fcimb.2022.805010 (PMC8960237; doi:10.3389/fcimb.2022.805010)
Supplement: Supplementary file 4 [file Table_3.docx]

**Supplementary table 3.** For *in* *vitro* co-cultural experiment, antibodies used for the flow cytometry analysis.

| Flow antibody | Dilution | Company | Cat | Labeled cells |
| --- | --- | --- | --- | --- |
| CD3-APC | 1:50 | Bioglend | 100236 | CD4^+^ T cells |
| CD4-FITC | 1:100 | Bioglend | 100406 |  |
|  |  |  |  |  |
| CD3-APC | 1:50 | Bioglend | 100236 | CD8^+^ Tcells |
| CD8-PE | 1:100 | ebioliscience | 12-0081-81 |  |
|  |  |  |  |  |
| CD3-APC | 1:50 | Bioglend | 100236 | Treg cells |
| CD4-FITC | 1:100 | Bioglend | 100406 |  |
| CD25-PE-Cy7 | 1:20 | BD | 552880 |  |
| CD127-PE | 1:20 | BD | 552543 |  |
|  |  |  |  |  |
| CD11b-FITC | 1:80 | Bioglend | 101206 | MDSC cells |
| Gr-1-PE | 1:80 | Bioglend | 108408 |  |
|  |  |  |  |  |
| CD19-PE | 1:50 | Bioglend | 115507 | B cells |

For uptake assay, we use PKH-67-FITC to label EVs, antibodies directed against the following makers were used to stain spleen mononuclear cells.

| Flow antibody | Dilution | Company | Cat | Label cells |
| --- | --- | --- | --- | --- |
| PKH-67-FITC |  | sigma | MIDI67-1KT | exosomes |
| CD3-PE | 1:50 | Biolegend | 100206 | CD4^+^ T cells |
| CD4-Percp | 1:100 | Biolegend | 100432 |  |
|  |  |  |  |  |
| CD3-Percp Cy5.5 | 1:50 | BD | 551163 | CD8^+^ T cells |
| CD8-PE | 1:100 | ebiolscience | 12-0081-81 |  |
|  |  |  |  |  |
| CD19-PE-Cy7 | 1:50 | Biolegend | 103209 | B cells |
|  |  |  |  |  |
| CD11b-PE-Cy5 | 1:80 | Biolegend | 101209 | MDSC |
| GR-1-PE | 1:80 | Biolegend | 108408 |  |
