# Supplementary material for: Proteomic Analysis of Plasma-Derived Extracellular Vesicles From Mice With Echinococcus granulosus at Different Infection Stages and Their Immunomodulatory Functions
Source: Front Cell Infect Microbiol. 2022 Mar 10;12:805010. doi: 10.3389/fcimb.2022.805010 (PMC8960237; doi:10.3389/fcimb.2022.805010)
Supplement: Supplementary file 5 [file Table_4.docx]

|  | total protein (mg/ml) | Concentration (particles/μg) |
| --- | --- | --- |
| 0W-EVs-1 | 1.00 | 4.8 × 10^8^ |
| 0W-EVs-2 | 1.10 | 4.6 × 10^8^ |
| 0W-EVs-3 | 1.05 | 4.5 × 10^8^ |
| 7W-EVs-1 | 1.30 | 4.3 × 10^8^ |
| 7W-EVs-2 | 1.20 | 4.5 × 10^8^ |
| 7W-EVs-3 | 1.25 | 4.3 × 10^8^ |
| 20W-EVs-1 | 1.30 | 4.7 × 10^8^ |
| 20W-EVs-2 | 1.23 | 4.4 × 10^8^ |
| 20W-EVs-3 | 1.26 | 4.2 × 10^8^ |

**Supplementary Table 4: The content of plasma EVs in mice infected with *E. granulosus* at different stages detected by bicinchoninic acid assay (BCA) and Nanoparticle tracking analysis (NTA).**
