# Supplementary material for: Proteomic Analysis of Plasma-Derived Extracellular Vesicles From Mice With Echinococcus granulosus at Different Infection Stages and Their Immunomodulatory Functions
Source: Front Cell Infect Microbiol. 2022 Mar 10;12:805010. doi: 10.3389/fcimb.2022.805010 (PMC8960237; doi:10.3389/fcimb.2022.805010)
Supplement: Supplementary file 6 [file Table_5.docx]

**Supplementary Table 5. Differential expressed proteins of plasma-EVs from mice infected with *E. granulosus* at different infection stages, containing uninfected (0W), 7-week post-infection (7W), 20-week post-infection (20W).** S0, S7 and S20 are used to represent the signal intensity of 0W-, 7W- and 20W- exosomes detected by mass spectrometry, respectively. A protein with a portion satisfying ratio greater than 1.5 or less than 1/1.5, and a *P* value < 0.05 is regarded as a different protein.

| Protein  accession | Gene  symbol | Protein description | Score | Unique  Peptides | Regulated Type | | |
| --- | --- | --- | --- | --- | --- | --- | --- |
|  |  |  |  |  | S20/S0 | S20/S7 | S7/S0 |
| Q9Z2W0 | Dnpep | Aspartyl aminopeptidase | 48.306 | 10 | Up |  | Up |
| Q9Z2U1 | Psma5 | Proteasome subunit alpha type-5 | 161.68 | 11 | Down |  | Down |
| Q9Z2U0 | Psma7 | Proteasome subunit alpha type-7 | 86.444 | 12 |  |  | Down |
| Q9Z1R3 | Apom | Apolipoprotein M | 14.993 | 6 | Down |  | Down |
| Q9Z126 | Pf4 | Platelet factor 4 | 21.603 | 5 |  |  | Down |
| Q9QZ25 | Vnn3 | Vascular non-inflammatory molecule 3 | 60.755 | 9 | Up |  | Up |
| Q9QWK4 | Cd5l | CD5 antigen-like | 323.31 | 29 | Down |  | Down |
| Q9ES30 | C1qtnf3 | Complement C1q tumor necrosis factor-related protein 3 | 25.273 | 5 | Up |  |  |
| Q9CQW3 | Proz | Vitamin K-dependent protein Z | 158.94 | 10 | Down |  | Down |
| Q91ZX7 | Lrp1 | Prolow-density lipoprotein receptor-related protein 1 | 190.99 | 33 |  | Up |  |
| Q91Y47 | F11 | Coagulation factor XI | 56.198 | 10 |  | Up |  |
| Q91WP6 | Serpina3n | Serine protease inhibitor A3N | 98.788 | 14 | Down |  |  |
| Q8VCM7 | Fgg | Fibrinogen gamma chain | 323.31 | 27 |  |  | Down |
| Q8VCG4 | C8g | Complement component C8 gamma chain | 147.37 | 11 | Up |  |  |
| Q8K1I3 | Spp2 | Secreted phosphoprotein 24 | 54.454 | 3 |  |  | Down |
| Q8CIZ8 | Vwf | von Willebrand factor | 68.012 | 18 |  |  | Up |
| Q8CG19 | Ltbp1 | Latent-transforming growth factor beta-binding protein 1 | 20.212 | 9 | Up |  | Up |
| Q8CF98 | Colec10 | Collectin-10 | 29.744 | 5 | Up |  | Up |
| Q8C7G5 | Apoa5 | Apolipoprotein A-V | 174.36 | 14 | Up |  | Up |
| Q8BTM8 | Flna | Filamin-A | 323.31 | 60 |  |  | Down |
| Q8BND5 | Qsox1 | Sulfhydryl oxidase 1 | 250.68 | 21 | Down |  | Down |
| Q6GQT1 | A2m | Alpha-2-macroglobulin-P | 323.31 | 63 | Up | Up | Up |
| Q64726 | Azgp1 | Zinc-alpha-2-glycoprotein | 87.72 | 18 | Down |  |  |
| Q61838 | Pzp | Pregnancy zone protein | 323.31 | 89 | Down |  |  |
| Q61268 | Apoc4 | Apolipoprotein C-IV | 46.369 | 6 | Down |  |  |
| Q60841 | Reln | Reelin | 151.72 | 25 | Up |  | Up |
| Q60692 | Psmb6 | Proteasome subunit beta type-6 | 41.723 | 6 | Down |  | Down |
| Q07797 | Lgals3bp | Galectin-3-binding protein | 181.37 | 13 |  |  | Down |
| Q06770 | Serpina6 | Corticosteroid-binding globulin | 183.38 | 18 |  | Down | Up |
| Q03734 | Serpina3m | Serine protease inhibitor A3M | 88.294 | 9 |  |  | Down |
| P70195 | Psmb7 | Proteasome subunit beta type-7 | 58.966 | 9 |  |  | Down |
| P52430 | Pon1 | Serum paraoxonase/arylesterase 1 | 110.82 | 12 | Down |  | Down |
| P51910 | Apod | Apolipoprotein D | 56.658 | 7 | Down |  | Down |
| P46412 | Gpx3 | Glutathione peroxidase 3 | 52.488 | 10 | Down |  | Down |
| P39876 | Timp3 | Metalloproteinase inhibitor 3 | 32.385 | 9 | Down |  | Down |
| P34928 | Apoc1 | Apolipoprotein C-I | 14.594 | 5 |  |  | Up |
| P33622 | Apoc3 | Apolipoprotein C-III | 88.189 | 4 | Up |  | Up |
| P31532 | Saa4 | Serum amyloid A-4 protein | 132.17 | 9 | Up |  |  |
| P28076 | Psmb9 | Proteasome subunit beta type-9 | 57.35 | 7 |  |  | Down |
| P26039 | Tln1 | Talin-1 | 323.31 | 65 |  |  | Down |
| P23953 | Ces1c | Carboxylesterase 1C | 137.93 | 12 |  | Down |  |
| P18532 | --- | Ig heavy chain V region 1B43 | 72.632 | 2 |  |  | Down |
| P18526 | --- | Ig heavy chain V region 345 | 73.114 | 3 |  |  | Down |
| P18525 | --- | Ig heavy chain V region 5-84 | 85.626 | 4 | Down |  | Down |
| P14847 | Crp | C-reactive protein | 87.477 | 9 | Down |  | Down |
| P13020 | Gsn | Gelsolin | 323.31 | 25 |  |  | Down |
| P11680 | Cfp | Properdin | 323.31 | 18 | Down |  | Down |
| P11276 | Fn1 | Fibronectin | 323.31 | 142 |  |  | Up |
| P08607 | C4bpa | C4b-binding protein | 203.65 | 20 | Down |  | Down |
| P08226 | Apoe | Apolipoprotein E | 323.31 | 29 | Up |  | Up |
| P07724 | Alb | Serum albumin | 323.31 | 69 |  | Down |  |
| P07309 | Ttr | Transthyretin | 230.13 | 9 |  |  | Up |
| P06683 | C9 | Complement component C9 | 170.45 | 24 | Up |  |  |
| P06330 | --- | Ig heavy chain V region AC38 205.12 | 91.568 | 3 | Down |  | Down |
| P06327 | Gm5629 | Ig heavy chain V region VH558 A1/A4 | 19.685 | 4 |  |  | Up |
| P04940 | --- | Ig kappa chain V-VI region NQ2-17.4.1 | 39.248 | 4 | Up |  | Up |
| P03987 | --- | Ig gamma-3 chain C region | 323.31 | 26 | Up |  | Up |
| P03976 | --- | Ig kappa chain V-II region 17S29.1 | 112.46 | 6 |  |  | Down |
| P01898 | H2-Q10 | H-2 class I histocompatibility antigen, Q10 alpha chain | 323.31 | 11 | Down |  |  |
| P01878 | --- | Ig alpha chain C region | 323.31 | 19 |  |  | Down |
| P01872 | Ighm | Ig mu chain C region | 323.31 | 35 | Up |  |  |
| P01867 | Igh-3 | Ig gamma-2B chain C region | 267.05 | 16 | Down |  | Down |
| P01843 | --- | Ig lambda-1 chain C region | 311.73 | 5 | Down |  | Down |
| P01820 | --- | Ig heavy chain V region PJ14 | 82.7 | 3 |  |  | Down |
| P01806 | --- | Ig heavy chain V region 441 | 190.2 | 2 | Up |  |  |
| P01790 | --- | Ig heavy chain V region M511 | 183.49 | 4 | Up |  | Up |
| P01783 | --- | Ig heavy chain V region MOPC 21 (Fragment) | 78.656 | 4 | Up | Up | Up |
| P01754 | Ighv1-62-3 | Ig heavy chain V region 1-62-3 | 13.173 | 0 | Up |  |  |
| P01725 | --- | Ig lambda-1 chain V region S178 | 8.8465 | 1 | Down |  | Down |
| P01670 | --- | Ig kappa chain V-III region PC 6684 | 159.76 | 0 | Down |  | Down |
| P01663 | --- | Ig kappa chain V-III region PC 4050 | 65.892 | 1 | Down |  |  |
| P01648 | --- | Ig kappa chain V-V region HP 91A3 | 8.4378 | 1 | Up |  | Up |
| P01645 | --- | Ig kappa chain V-V region HP 93G7 | 83.168 | 1 | Up |  | Up |
| P01643 | --- | Ig kappa chain V-V region MOPC 173 | 46.33 | 1 | Up |  |  |
| P01027 | C3 | Complement C3 | 323.31 | 140 | Up |  | Up |
| O88947 | F10 | Coagulation factor X | 323.31 | 18 | Down |  |  |
| O70570 | Pigr | Polymeric immunoglobulin receptor | 108.55 | 15 | Down |  | Down |
| O08677 | Kng1 | Kininogen-1 | 323.31 | 26 | Down |  | Down |
| B2RPV6 | Mmrn1 | Multimerin-1 | 323.31 | 33 |  | Up | Down |
